# mTOR Repression in Response to Amino Acid Starvation Promotes ECM Degradation Through MT1‐MMP Endocytosis Arrest

**DOI:** 10.1002/advs.202101614

**Published:** 2021-07-11

**Authors:** Cecilia Colombero, David Remy, Sandra Antoine‐Bally, Anne‐Sophie Macé, Pedro Monteiro, Nadia ElKhatib, Margot Fournier, Ahmed Dahmani, Elodie Montaudon, Guillaume Montagnac, Elisabetta Marangoni, Philippe Chavrier

**Affiliations:** ^1^ Institut Curie PSL Research University CNRS UMR 144 Paris 75005 France; ^2^ Cell and Tissue Imaging Facility (PICT‐IBiSA) Institut Curie PSL Research University Paris 75005 France; ^3^ Gustave Roussy Institute Université Paris‐Saclay INSERM U1279 Villejuif 94805 France; ^4^ Translational Research Department Institut Curie PSL Research University Paris 75005 France

**Keywords:** breast cancer, clathrin‐mediated endocytosis, extracellular matrix, invadopodia, MT1‐MMP, mTOR, starvation

## Abstract

Under conditions of starvation, normal and tumor epithelial cells can rewire their metabolism toward the consumption of extracellular proteins, including extracellular matrix‐derived components as nutrient sources. The mechanism of pericellular matrix degradation by starved cells has been largely overlooked. Here it is shown that matrix degradation by breast and pancreatic tumor cells and patient‐derived xenograft explants increases by one order of magnitude upon amino acid and growth factor deprivation. In addition, it is found that collagenolysis requires the invadopodia components, TKS5, and the transmembrane metalloproteinase, MT1‐MMP, which are key to the tumor invasion program. Increased collagenolysis is controlled by mTOR repression upon nutrient depletion or pharmacological inhibition by rapamycin. The results reveal that starvation hampers clathrin‐mediated endocytosis, resulting in MT1‐MMP accumulation in arrested clathrin‐coated pits. The study uncovers a new mechanism whereby mTOR repression in starved cells leads to the repurposing of abundant plasma membrane clathrin‐coated pits into robust ECM‐degradative assemblies.

## Graphical abstract

Cancer cells catabolize extracellular matrix (ECM) components to maintain growth when nutrients are depleted. This study reports that tumor cells repurpose the invadopodia‐, protease‐dependent program to elicit a massive ECM degradation mechanism, under regulation by mechanistic target of rapamycin complex 1. Nutrient scarcity blocks endocytosis of membrane type 1‐matrix metalloproteinase converting arrested clathrin‐coated pits into powerful matrix degradation units.

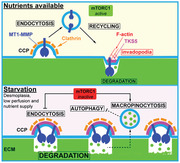

## 1 Introduction

Metabolic reprogramming is a hallmark of cancer cells, which adapt their nutritional strategies to match their elevated metabolic needs.^[^ ^1^, ^2^ ^]^ In certain microenvironments including in poorly perfused tumors, free nutrients such as amino acids (AAs) can be limiting, and extracellular proteins are used as alternative resources.^[^ ^3^, ^4^, ^5^, ^6^, ^7^ ^]^ Recent studies found that in desmoplastic microenvironments, pancreatic and breast cancer cells can internalize proteolytic extracellular matrix (ECM) fragments such as peptides derived from fibronectin and type I collagen, which accounts for most of the extracellular biomass in these tumors.^[^ ^8^, ^9^, ^10^, ^11^ ^]^ Degradation of ECM‐derived peptides in lysosomes contributes to AA supply that fuels the tumor metabolism and supports tumor survival and proliferation.^[^ ^10^, ^11^, ^12^ ^]^ In addition, high collagen density has been linked with metabolism rewiring in breast cancer cells.^[^ ^7^, ^13^ ^]^ However, the mechanism underlying ECM breakdown under nutrient‐depleted conditions is unknown.

The cell response to nutrients is controlled by the kinase mechanistic target of rapamycin (mTOR), which assembles into distinct protein complexes known as mTOR Complex 1 and 2 (mTORC1 and ‐2).^[^ ^14^ ^]^ Only mTORC1 is sensitive to acute treatment by the anticancer drug, rapamycin.^[^ ^14^ ^]^ Under AA replete conditions, mTORC1 localizes to the lysosome surface and phosphorylates several substrates including S6K and 4E‐BP1, promoting protein translation and cell growth. Upon AA starvation, mTORC1 is inactivated inducing autophagy, cellular catabolism, and translation shut down.^[^ ^14^ ^]^ Dysregulation of the PI3K/Akt/mTOR signaling pathway is linked with breast cancer initiation and progression and regional heterogeneity in immunohistochemical profiles of phosphorylated (p)‑mTOR and its downstream signaling effectors, pS6K, and p4E‐BP1 have been described in relation with metabolic alterations.^[^ ^15^, ^16^ ^]^

The protease‐dependent invasion program of tumor cells is mediated by invadopodia, which are F‐actin‐, cortactin‐based cell–matrix contacts that enzymatically degrade and push confining ECM fibers aside to allow cell movement.^[^ ^17^, ^18^, ^19^, ^20^ ^]^ The scaffolding protein, TKS5, plays a pivotal role in the assembly and surface accumulation of the trans‐membrane matrix metalloproteinase and collagenase, MT1‐MMP, to invadopodia.^[^ ^20^, ^21^, ^22^, ^23^ ^]^ Here, we investigated the mechanism of ECM degradation under conditions of nutrient scarcity in relation with mTOR signaling. Our findings uncover a novel mechanism that leads to the repurposing of the invadopodial MT1‐MMP/TKS5 axis. This program, which is controlled by mTOR signaling, inhibits the endocytic clearance of MT1‐MMP and triggers its accumulation in arrested plasma membrane clathrin‐coated pits (CCPs) to actively degrade the collagen matrix in an AA‐depleted environment.

## 2 Results

### 2.1 Starvation of Tumor Cells Stimulates Invadopodia‐Mediated Pericellular Matrix Degradation

**Figure 1:**
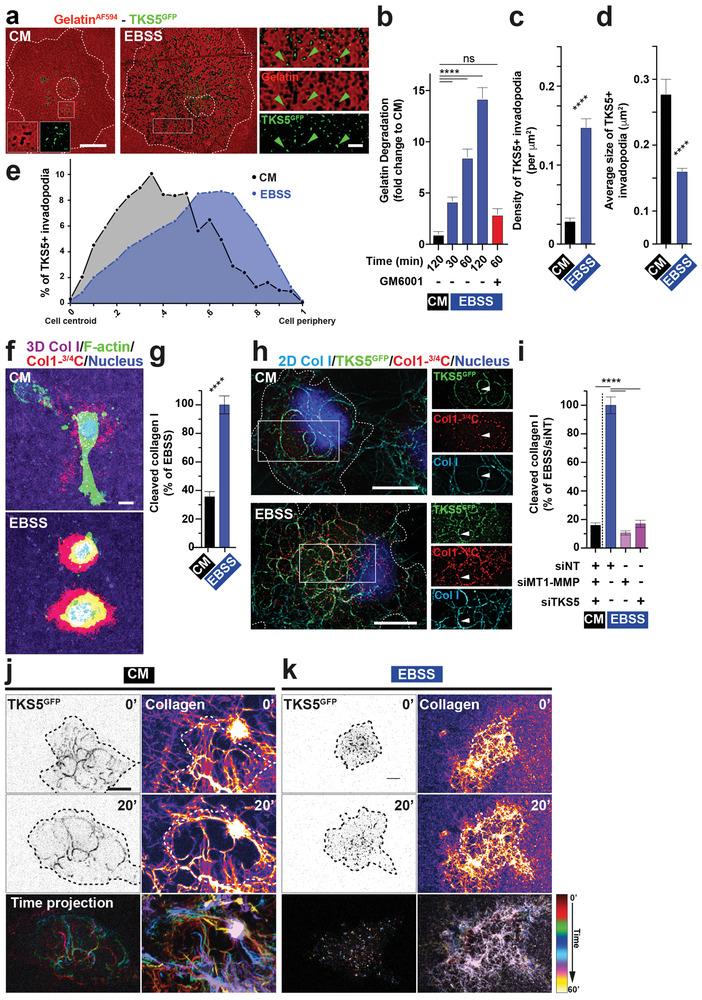
Enhanced matrix degradation and requirement for MT1‐MMP and TKS5 in AA‐ and serum‐starved cells. a) Deconvoluted images showing MDA‐MB‐231 cells expressing TKS5^GFP^ (green) plated on fluorescently‐labeled gelatin (red) for 60 min in CM or EBSS starvation medium depleted for AAs and serum. Higher magnification of the boxed region is shown in the insets. Dotted lines, cell and nucleus contour. Scale bars, 10 µm, 2 µm (insets). b) Gelatin degradation by MDA‐MB‐231 cells cultured in the indicated conditions. c) Mean density of TKS5‐positive invadopodia ± SEM (invadopodia µm^‐2^). d) Mean size of TKS5‐positive invadopodia ± SEM (µm^2^). e) Mean percentage distribution of TKS5‐positive invadopodia according to their cell center‐to‐cell periphery (0‐1) position in MDA‐MB‐231 cells cultured in the indicated conditions. f) MDA‐MB‐231 cells embedded in a 3D collagen I gel (magenta) for 6 h in the indicated medium and stained for cleaved collagen (red); F‐actin (green); nucleus (blue). Scale bar, 10 µm. g) Collagen degradation by MDA‐MB‐231 cells cultured in CM or EBSS media normalized to EBSS value. h) Deconvoluted images showing MDA‐MB‐231 cells expressing TKS5^GFP^ cultured on a fibrillar type I collagen network (cyan) for 60 min in indicated medium and stained for TKS5^GFP^ (green), cleaved collagen fibers (red), and nucleus (blue). Higher magnification of boxed regions is shown in the insets. Arrowheads, TKS5^GFP^‐positive invadopodia. Scale bar, 10 µm. i) Collagen degradation by MDA‐MB‐231 cells treated with the indicated siRNAs. j,k) MDA‐MB‐231 cells expressing TKS5^GFP^ were cultured on fluorescently‐labeled type I collagen in CM or EBSS medium and imaged over time (1 image min^−1^) by video microscopy (see Movies S1 and S2, Supporting Information). The first and 20^th^ images of representative time‐lapse sequences are displayed in the upper left row showing TKS5^GFP^‐positive invadopodia using an inverted grayscale lookup table. The upper right row shows the collagen network using a Fire lookup table. The dotted lines underline the cell contour. The bottom row displays color‐coded time projections of seven images at 10‐min intervals showing the dynamics of TKS5^GFP^‐positive invadopodia and the remodeling of type I collagen fibers over time.

MDA‐MB‐231 cells were selected as a model of breast cancer cells known for producing a robust invadopodial response at matrix contact sites.^[^ ^20^ ^]^ When plated on fluorescently labeled gelatin for 60 min in complete medium (CM), several gelatin degradation spots were visible underneath the cells, which coincided with punctate invadopodia positive for TKS5 (endogenous or overexpressed GFP‐tagged protein) located underneath or in the vicinity of the nucleus (**Figure** **1a** and Figure S1a, Supporting Information). The consequences of starvation on ECM degradation were assessed by culturing cells in AA‐ and serum‐depleted medium (EBSS). Starvation robustly increased the degradation of gelatin by MDA‐MB‐231 cells (Figure 1a,b and Figure S1a, Supporting Information). This response was abolished by treatment with the broad‐spectrum MMP inhibitor, GM6001 (Figure 1b). Increased gelatinolysis correlated with a 4‐5‐fold increase in the density of matrix‐degradative TKS5‐positive structures, which were reduced in size as compared with those in cells incubated in CM (Figure 1c,d). Analysis of the invadopodia distribution along a cell centroid‐to‐periphery axis (0‐1 position) revealed that invadopodia were homogeneously scattered throughout the entire adherent cell surface in starved cells in contrast to their typical central localization in cells in replete conditions (Figure 1e). In order to exclude that association of TKS5‐positive dotty structures with degradation spots was by chance due to the high structure density in starved cells, TKS5 positions were randomly scrambled 5000 times, and the association of TKS5‐positive structures with degradation spots was calculated for each scrambled image (Figure S1b,c, Supporting Information). The observed association values exceeded all those calculated for randomly scrambled images ruling out that TKS5 association with degradation spots was random (Table S5, Supporting Information).

Cells were embedded within a 3D fibrillar collagen network, the main component of interstitial ECM tissue, and stained with a Col1‐¾C antibody that recorded collagen cleavage cumulated over the incubation period. Similar to the gelatinolysis response, collagen cleavage by starved cells was strongly enhanced as compared to replete conditions (Figure 1f,g). Although the association of the invadopodia components, cortactin, and TKS5, with cleaved fibers was visible irrespective of nutrient availability, invadopodia occupied a much larger portion of the cell surface and were more fragmented in starved cells (Figure 1h and Figure S1d, Supporting Information). MT1‐MMP can be inhibited by tissue inhibitors of metalloproteinases (TIMPs), including TIMP‐2 which is present in tissues and biological fluids including serum.^[^ ^24^ ^]^ EBSS medium was supplemented with increasing amount of recombinant TIMP‐2 from two independent sources. Only at the dose of 2 µg mL^−1^ (i.e., 20‐200‐fold TIMP‐2 concentration in CM, not shown),^[^ ^24^ ^]^ was recombinant human TIMP‐2 capable of fully repressing collagenolysis (Figure S1e, Supporting Information). These data show that the absence of TIMP‐2 in EBSS condition does not account for the observed changes in matrix degradation by starved cells. Moreover, it is likely that TIMP‐2 concentration is reduced in tumors embedded within a dense collagen‐rich desmoplasia lowering vascularization.

As reported, silencing of MT1‐MMP or TKS5 inhibited collagen cleavage in MDA‐MB‐231 cells in replete conditions (Figure S1f,g, Supporting Information).^[^ ^23^ ^]^ Depletion of MT1‐MMP or TKS5 also abolished collagenolysis in starved cells (Figure 1i). MT1‐MMP was similarly required for induction of gelatinolysis in EBSS (Figure S1h, Supporting Information). Induction of collagenolysis upon starvation and MT1‐MMP and TKS5 dependency were seemingly observed in pancreatic adenocarcinoma Bx‐PC3 cells, which expressed levels of the main invadopodia components similar to breast MDA‐MB‐213 cells (Figure S2a,d, Supporting Information). Collectively, these findings indicate that increased matrix degradation by AA‐depleted cells is mediated by a MT1‐MMP and TKS5‐dependent mechanism in breast and pancreatic tumor cells.

We compared the capacity of cells to remodel collagen fibers in nutrient replete or deplete environments by live cell imaging. TKS5^GFP^‐positive invadopodia in cells in replete conditions were highly dynamic and were able to push aside and bundled the contacted collagen fibers (Figure 1j and Movie S1, Supporting Information).^[^ ^20^ ^]^ In contrast, fragmented and mostly static TKS5‐positive structures were visible in AA‐starved cells with the little remodeling of the underlying fibers over time (Figure 1k and Movie S2, Supporting Information). These observations demonstrate that tumor cells switch from a potent matrix remodeling and invasive mode in replete conditions typical of disseminating cells, to an exclusive matrix degradation and possibly nutrient sourcing program in nutrient‐scarce conditions.

### 2.2 Induction of the MT1‐MMP Collagenolytic Response Upon Starvation of TNBC PDX Explants

**Figure 2:**
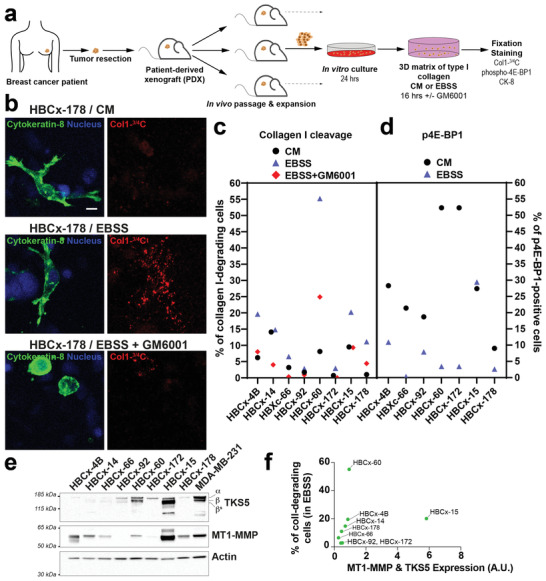
Induced collagenolysis in starved TNBC PDX ex vivo. a) Scheme depicting the preparation and analysis of breast cancer‐derived PDX explants. b) PDX cells embedded in type I collagen in the indicated culture conditions were fixed and epithelial breast tumor cells were stained for Cytokeratin‐8 (green) and cleaved collagen (red). DAPI‐stained nuclei are shown in blue. Scale bar, 10 µm. c) Collagen cleavage by epithelial breast tumor cells derived from PDXs cultured in CM or EBSS medium in the presence or absence of GM6001. d) Levels of phosphorylated (p)4E‐BP1 in epithelial breast tumor cells derived from PDXs cultured in CM or EBSS medium. e) Representative western blots of TKS5 and MT1‐MMP expression in the PDX‐derived cells grown in complete medium. Actin was used as a loading control. Molecular weights are in kDa. f) Levels of MT1‐MMP and TKS5 expression in PDXs normalized to F‐actin based on immunoblotting analysis show in panel d were summed up (*x*‐axis) and plotted vs. the percentage of collagen degrading‐cells (*y*‐axis) for each starved PDX.

To generalize these findings to a model close to the human disease, we used cells obtained from triple‐negative breast cancer patient‐derived xenografts (PDX).^[^ ^25^, ^26^ ^]^ Cells isolated from several independent PDXs were cultured *ex vivo* in a 3D type I collagen gel in AA‐replete or depleted conditions and their collagenolytic activity was assessed (**Figure** **2a**). PDX‐derived cells were primarily composed of cytokeratin‐8‐positive carcinoma cells and fibroblasts, the latter being identified based on their characteristic spindle‐shaped morphology (not shown). In five out of eight PDXs, overnight incubation in EBSS resulted in a strong induction of pericellular collagen degradation as compared to the complete medium (Figure 2b,c). Moreover, treatment with GM6001 inhibited collagenolysis supporting the conclusion that stimulation of ECM degradation requires MMP activity in starved PDXs (Figure 2d). Starvation of the PDXs correlated with reduced phosphorylated 4E‐BP1 (Ser65) level as a proxy for mTORC1 kinase activity as compared to conditions of nutrient sufficiency (Figure 2d). Additionally, we found a correlation between the intensity of the starvation‐induced collagenolytic response with MT1‐MMP, and to some extent TKS5, expression levels in PDX (for instance compare the response of TKS5^Low^ and MT1‐MMP^Low^ HBCx‐66, HBCx‐92 and HBCx‐172 and MT1‐MMP^High^ HBCx‐4B and HBCx‐60 PDXs, Figure 2e,f). Collectively, these data support the conclusion that nutrient scarcity enhances collagenolysis in breast PDXs in relation with the TKS5/MT1‐MMP axis.

### 2.3 Regulation of the Collagenolytic Response by mTOR Signaling

**Figure 3:**
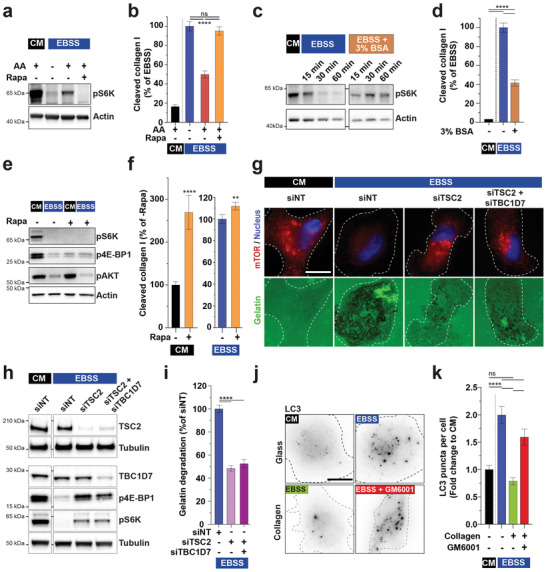
Regulation of ECM degradation by mTORC1. a,b) Phosphorylated (p)S6K (Thr389) in MDA‐MB‐231 cells incubated for 60 min in CM or EBSS medium supplemented with AA in the presence or absence of rapamycin. Actin was used as a loading control (panel a). Collagen cleavage by MDA‐MB‐231 cells incubated in the indicated conditions (panel b). c) Levels of pS6K in MDA‐MB‐231 cells incubated for the indicated period of time in CM or EBSS medium supplemented with 3% BSA. d) Collagen cleavage by MDA‐MB‐231 cells incubated for 60 min in the indicated medium. e) Immunoblots of total and phosphorylated S6K, 4E‐BP1 (Ser65), and AKT (Ser473) in MDA‐MB‐231 cells incubated for 60 min in the indicated medium in the presence of mTOR inhibitor, rapamycin, or with the corresponding vehicle with actin used as a loading control. f) Collagen cleavage by MDA‐MB‐231 cells incubated for 60 min in indicated medium with or without rapamycin. g) MDA‐MB‐231 cells treated with the indicated siRNAs, were plated on fluorescently‐labeled gelatin (green) for 60 min in CM or EBSS starvation medium. Cells were fixed and analyzed by immunofluorescence staining for mTOR (red). The cell contour is shown with a dotted line. Scale bar, 10 µm. h) Immunoblots of TSC2, TBC1D7, p4E‐BP1, and pS6K in MDA‐MB‐231 cells incubated for 60 min in the indicated medium. Tubulin was used as a loading control. i) Gelatin degradation by MDA‐MB‐231 cells cultured in the indicated conditions. j,k) Quantification of autophagy LC3 puncta in MDA‐MB‐231 cells cultured in the indicated conditions normalized to the mean value in cells grown in CM medium on plastic ± SEM. Scale bar, 10 µm.

mTOR is the master regulator of the cell's response to nutrient and AA availability.^[^ ^14^ ^]^ As expected, mTORC1 activity was strongly repressed in cells cultured for 1 h in EBSS as compared to CM as shown by the reduction in phosphorylated S6K (Thr389) and p4E‐BP1 levels (**Figure** **3a**,e,h). Replenishment of EBSS with free AAs similar to their concentration in CM (see Table S4, Supporting Information) partially restored mTORC1 activity (Figure 3a), and was correlated with a 50–60% reduction of collagen cleavage by MDA‐MB‐231 cells as compared to EBSS (Figure 3b). These findings indicated that nutrient scarcity, in particular the lack of free AAs, strengthens the collagenolytic activity of breast MDA‐MB‐231 tumor cells, and that the absence of serum components accounted for ≈50% of the response. In addition, it has been shown that oncogenic Ras mutations promote macropinocytic uptake of extracellular proteins such as serum albumin, which are catabolized in lysosomes and serve as an AA source to sustain cancer cells’ metabolic needs.^[^ ^3^, ^4^, ^27^ ^]^ We observed that supplementing EBSS with 3% bovine serum albumin (BSA) partially restored pS6K levels in agreement with the mutated *KRAS* status of MDA‐MB‐231 cells (Figure 3c, and Figure S3a, Supporting Information),^[^ ^28^, ^29^ ^]^ and resulted in a ≈60% reduction of collagen cleavage as compared to EBSS alone (Figure 3d). Alltogether, these data suggest a correlation between mTORC1 activity and ECM degradation, i.e., mTORC1 inhibition correlates with the induction of matrix degradation by breast tumor cells.

In agreement with this assumption, we observed that inhibition of mTORC1 activity by acute rapamycin treatment of cells grown in nutrient‐replete conditions (CM) (Figure 3e, and Figure S3b–d, Supporting Information), resulted in a ≈2.5‐4‐fold increase in collagen or gelatin degradation (Figure 3f and Figure S3e, Supporting Information). In contrast, collagenolysis was only marginally increased upon rapamycin treatment in EBSS (Figure 3f), in conjunction with the fully repressed mTORC1 status in starved cells (Figure 3e, and Figure S3b–d, Supporting Information). Interestingly, reduced collagenolysis upon EBSS supplementation with free AAs was abolished in cells treated with rapamycin in parallel with fully repressed mTORC1 activity (Figure 3a,b). mTORC1 integrates convergent AA‐sensing signals from Rag and signaling inputs from Rheb GTP‐binding proteins on endolysosomes.^[^ ^14^ ^]^ The TSC complex (TSC1, TSC2, and TBC1D7), which acts as a GTPase activating protein (GAP) for Rheb, is recruited to the endolysosomes by Rag GTPases upon AA removal causing Rheb inactivation.^[^ ^30^ ^]^ Expectedly, we observed a redistribution of mTOR from perinuclear vesicular compartments in CM to a diffuse cytosolic mTOR staining along with the disappearance of p4E‐BP1 and pS6K signals in starved cells consistent with endolysosome dissociation and repression of mTOR (Figure 3g,h). In agreement with previous observations,^[^ ^30^ ^]^ we found that starved cells silenced for TSC2 and TBC1D7 subunits failed to completely inactivate mTORC1 as shown by residual mTOR association with perinuclear membrane compartments and detection of pS6K and p4E‐BP1 signal (Figure 3g,h). TSC2 and TBC1D7 knockdown led to approximately twofold reduction of gelatinolysis in EBSS that strongly correlated with some persistence of active mTOR on perinuclear endolysosomes (Figure 3g,i). Collectively, these data are consistent with a direct control of ECM degradation by mTORC1 activity.

In order to further strengthen the interplay between mTORC1 activity and the ECM degradation response, autophagy levels were investigated in starved cells in the absence or presence of type I collagen by staining for the autophagy marker, LC3.^[^ ^31^ ^]^ As expected, LC3‐positive vesicular structures increased upon starvation of MDA‐MB‐231 cells cultured on plastic as compared to replete conditions (Figure 3j,k). Interestingly, autophagy was significantly reduced in starved cells cultured in the presence of type I collagen, and this effect was partially abrogated upon MMP inhibition by GM6001 treatment (Figure 3j,k). All together, these results confirm that AA scarcity represses mTORC1 activity leading to the induction of autophagy and show that the autophagy response is inhibited by type I collagen in the presence of active MMP (presumably MT1‐MMP). These data suggest that collagen breakdown by MT1‐MMP may produce AA resources,^[^ ^10^, ^11^ ^]^ which restore some level of mTOR activity leading to the downmodulation of the autophagy response in starved MDA‐MB‐231 cells.

### 2.4 Endocytic Arrest and CCP Retention of MT1‐MMP in Starved Cells

**Figure 4:**
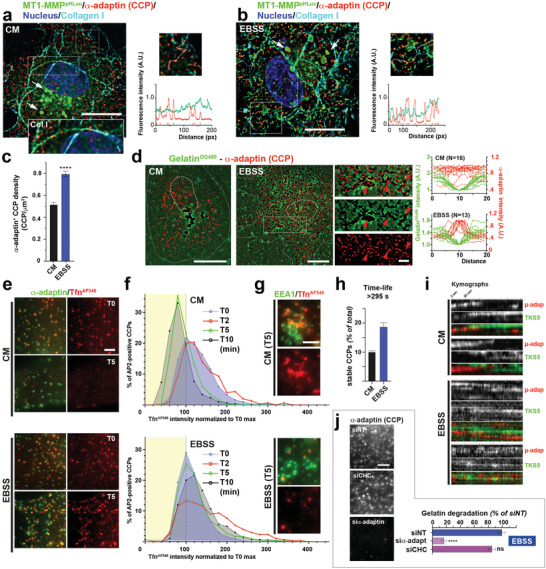
Starvation induces MT1‐MMP endocytic arrest. a,b) Deconvoluted images showing the distribution of MT1‐MMP^pHLuorin^ (green) and *α*‐adaptin‐positive CCPs (red) in MDA‐MB‐231 cells cultured on fibrillar type I collagen (cyan) in the indicated medium. DAPI‐stained nuclei are shown in blue. White arrows, fluorescence signal of MT1‐MMP^pHLuorin^ in endolysosomes visible after cell fixation. Inset shows higher magnification of the boxed region. Scale bar, 10 µm. Right panels, intensity profile (linescan) of *α*‐adaptin and MT1‐MMP^pHLuorin^ signals (red arrows, CCPs). c) Mean density of *α*‐adaptin‐positive CCPs ± SEM (CCP µm^‐2^). d) Deconvoluted images showing the distribution of *α*‐adaptin‐positive CCPs (red) in cells plated on fluorescently‐labeled gelatin (green) in indicated medium. Dotted lines, cell and nucleus contour. Scale bars, 10 µm; 2 µm (insets). Red arrowheads, CCP associated with gelatin degradation spots. Right panels, linescan intensity profiles of *α*‐adaptin (red) and gelatin fluorescence in degraded areas. e) Fluorescence signal of Tfn^AF546^ (red) associated with *α*‐adaptin‐positive CCPs (green) in cells cultured in the absence of labeled Tfn in the indicated medium. Scale bar, 2 µm. f) Kinetics of Tfn^AF546^ uptake in MDA‐MB‐231 cells incubated in the indicated medium. g) Internalization of Tfn^AF546^ (red) in EEA1‐enriched endosomes (green) is reduced upon cell starvation. Scale bar, 2 µm. h) Percentage of stable CCPs ± SEM (lifetime > 295 s). i) Kymographs showing CCP and TKS5 dynamics in cells expressing *μ*‐adaptin^mCh^ and TKS5^GFP^ plated on unlabeled gelatin in the indicated medium. Cells were imaged by TIRF‐M every 5 s for 5 min. j) Gelatinolysis by starved cells silenced for *α*‐adaptin or CHC. Right panels show *α*‐adaptin‐positive CCPs in the different cell populations. Scale bar, 2 µm.

Total levels of MT1‐MMP remained steady for at least 6 h in starved cells suggesting some redistribution of a preexisting pool to support the increase in collagenolysis (Figure S4a, Supporting Information). The influence of nutrient availability on the distribution of MT1‐MMP was analyzed. Confirming previous observations, MT1‐MMP fused with a GFP variant (pHLuorin)‐tag localized predominantly in perinuclear late endosomes/lysosomes from which it can recycle to plasma membrane invadopodia (**Figure** **4a**,b).^[^ ^32^ ^]^ In addition, in cells grown in replete conditions, MT1‐MMP was detected in plasma membrane accumulations in association with the underlying collagen fibers (i.e., invadopodia, Figure 4a). In contrast, MT1‐MMP had an extensive dotty‐like surface distribution in cells cultured in EBSS (Figure 4b). Endocytic CCPs cover the entire cell surface and display an archetypical dotty distribution. Moreover, the LLY^573^ motif in the carboxy‐terminal tail of MT1‐MMP is known to interact with the clathrin adaptor AP‐2 complex involved in MT1‐MMP surface clearance (see below).^[^ ^33^ ^]^ Counterstaining for the *α*‐adaptin subunit of AP‐2 revealed that MT1‐MMP‐positive puncta were in close proximity to CCPs in starved cells (Figure 4b). Additionally, we noticed a 1.6‐fold increase in the density of CCPs at the plasma membrane of starved cells as compared to cells grown in CM (Figure 4c).

Similarly, we observed a striking association of gelatin degradation spots and AP‐2‐positive CCPs in cells plated on fluorescently‐labeled gelatin matrix grown in EBSS medium (Figure 4d, right panel and Figure S4b,c, Supporting Information). MT1‐MMP punctate accumulations also coincided with the degradation spots (Figure S4d, Supporting Information). All together, accumulations of TKS5 (Figure 1a), AP‐2 (Figure) and MT1‐MMP (Figure S4d, Supporting Information) in association with a prominent dotty matrix degradation pattern appear as a strong emerging feature of starved cells.

Constitutive endocytosis of the transferrin (Tfn)‐receptor is mediated by clathrin and AP‐2 and can be readily monitored using fluorophore‐conjugated Tfn. We followed the decay of Tfn^AF546^ from *α*‐adaptin‐positive CCPs overtime in cells cultured in CM or EBSS as a quantification of clathrin‐mediated endocytosis (CME). While CCP‐associated Tfn^AF546^ rapidly decayed in cells incubated in CM medium in the absence of fluorescent ligand, the intensity of receptor‐bound Tfn^AF546^ associated with CCPs remained almost constant over the 10 min pulse in EBSS (Figure 4e,f). Internalized Tfn^AF546^ rapidly reached EEA1‐positive early endosomes in cells incubated in nutrient‐replete conditions, while the amount of Tfn^AF546^ detected in early endosomes was much lower in starved cells consistent with the reduction in Tfn uptake (Figure 4g). Additionally, we followed the dynamics of µ‐adaptin^mCh^‐positive CCPs by TIRF‐M and found approximately twofold increase in the percentage of stable CCPs (lifetime > 295 s) in EBSS versus CM conditions (Figure 4h and Movie S3, Supporting Information), in agreement with the observed increase in CCP density and reduced CME flux. Interestingly, TIRF‐M also revealed some association between CCPs and TKS5^GFP^‐positive puncta that formed in their vicinity (Figure 4j and Movie S3, Supporting Information). Similar to stable CCPs in starved cells, adjacent TKS5^GFP^‐positive puncta also appeared to be long‐lived (Figure 4i, and Movie S3, Supporting Information). Finally, we found that under conditions of endocytic arrest in starved cells, the induction of gelatinolysis was abrogated by *α*‐adaptin knockdown (Figure 4j and Figure S4e, Supporting Information). This was in sharp contrast to the silencing of clathrin heavy chain that did not significantly impair matrix degradation nor AP‐2 cluster formation (Figure 4j). All together, these observations highlight the requirement for the clustering of surface‐exposed MT1‐MMP to sustain the starvation‐induced ECM degradation response through a mechanism, which, likely, involves the interaction of MT1‐MMP with AP‐2 in arrested CCPs (see Figure S4f, Supporting Information).^[^ ^33^ ^]^

## 3 Discussion and Conclusion

We show that depletion of extracellular AAs and serum to replicate conditions of nutrient scarcity in a collagen‐rich microenvironment elicits a robust cancer cell‐autonomous collagenolytic response, exceeding by one‐order‐of‐magnitude the ECM‐degradative activity of invasive breast and pancreatic cell lines and breast PDXs. The pericellular ECM‐degradation response to starvation is triggered by mTOR inactivation and we identified the key invadopodia components, TKS5 and MT1‐MMP, as major players. In contrast to its association to dynamically forming invadopodia at ECM contact sites typical of invasive cells under nutrient‐replete conditions,^[^ ^19^, ^20^ ^]^ surface‐exposed MT1‐MMP accumulates at arrested CCPs in cells in a nutrient‐scarce environment. Intriguingly, we observed some association between dynamic TKS5‐positive assemblies and CCPs, which is enhanced upon starvation. Interestingly, several CME regulators including inositol 5‐phosphatase, SHIP2, and its product, phosphatidylinositol 3,4‐bisphosphate (PI(3,4)P2), the F‐BAR domain proteins, CIP4 and FBP17, the Arp2/3 complex activator, N‐WASP, and cortactin are known TKS5 interactors involved in invadopodia formation, suggesting that related mechanisms operate at CCPs and invadopodia.^[^ ^23^, ^34^, ^35^, ^36^ ^]^ Interaction of TKS5 with stable CCPs in conjunction with MT1‐MMP clustering based on binding to the AP‐2 clathrin‐adaptor complex is probably key to the repurposing of CCPs into powerful ECM‐degradative assemblies.

A dual role for collagenolytic invadopodia has been found during tumor cell invasion.^[^ ^20^ ^]^ On the one hand, limited proteolysis of individual collagen molecules by invadopodial MT1‐MMP can soften the fibrils to facilitate cell passage during confined invasion.^[^ ^20^ ^]^ On the other hand, invadopodia can generate outward forces to push collagen fibers aside using the energy of actin polymerization.^[^ ^20^, ^37^ ^]^ Although CCPs have been found to form in association with and can grab collagen fibers,^[^ ^38^ ^]^ actin‐based forces generated at CCPs are inwardly oriented to facilitate the budding of endocytic clathrin‐coated vesicles.^[^ ^39^ ^]^ Thus, it is unlikely that CCPs could exert pushing forces on matrix fibers. It is more plausible that the approximately 10‐fold increase in collagenolysis under nutrient restriction conditions leads to the fragmentation of ECM fibers facilitating their internalization and incorporation in the cell metabolism.^[^ ^10^, ^11^, ^12^ ^]^ It is thus tempting to speculate that MT1‐MMP‐rich CCPs in starved cells have limited impact on tumor cell invasion, rather transforming the entire plasma membrane into an ECM‐degradative surface and promoting a vigorous nutrient sourcing program.

Earlier studies in cell lines and in drosophila and mouse models revealed that genetic or pharmacological inhibition of mTOR kinase impedes endocytosis, similar to the observations.^[^ ^40^, ^41^, ^42^, ^43^ ^]^ Other reports highlighted that nutrient scarcity and mTORC1 inhibition stimulate the nutritional use of extracellular proteins and that combined mTORC1 and ‐2 inhibition induces macropinocytosis, the main route for extracellular protein uptake by cancer cells.^[^ ^3^, ^4^, ^5^, ^11^, ^27^, ^44^ ^]^ Collectively, despite mechanistic details that are missing, these data point to some opposite effects of starvation and mTOR inhibition on the downmodulation of CME and activation of macropinocytic (or related phagocytic) uptake, which could cooperate in the production and internalization of ECM fragments by tumor cells. The study suggests the extreme capacity of cancer cells to rewire their nutritional plans and metabolism for survival and growth in adverse conditions by repurposing an ECM proteolysis machinery. It also underscores potential limitations of anti‐mTOR therapeutic strategies as mTOR inhibition can unleash the ECM‐degradative potential of carcinoma cells.

## Conflict of Interest

The authors declare no conflict of interest.

## Author Contributions

C.C. and D.R. contributed equally to this work. C.C. and D.R.: conceptualization, investigation, methodology, validation, formal analysis, manuscript editing. S.A‐B., P.M., and N.E.: methodology, investigation, validation, analysis. A.‐S.M.: image processing and analysis. M.F., A.D., and E.M.: methodology. G.M.: supervision of TIRF‐M analysis. E.M.: supervision of PDX experiments. P.C.: conceived the study, conceptualization, validation, visualization, drafting of the manuscript, funding acquisition, supervision, and project administration.

## Supporting information

Supporting Information

Supplemental Movie 1

Supplemental Movie 2

Supplemental Movie 3

## Data Availability

Data available on request from the authors.
