# Supplementary material for: mTOR Repression in Response to Amino Acid Starvation Promotes ECM Degradation Through MT1‐MMP Endocytosis Arrest
Source: Adv Sci (Weinh). 2021 Jul 11;8(17):2101614. doi: 10.1002/advs.202101614 (PMC8425857; doi:10.1002/advs.202101614)
Supplement: Supplementary file 1 — Supporting Information [file ADVS-8-2101614-s002.pdf]

© 2021 Wiley-VCH GmbH

ADVANCED SCIENCE
Open Access

Supporting Information

for *Adv. Sci.,* DOI: 10.1002/advs.202101614

mTOR repression in response to amino acid starvation promotes ECM degradation through MT1-MMP endocytosis arrest

*Cecilia Colombero, David Remy, Sandra Antoine-Bally, Anne-Sophie Macé, Pedro Monteiro, Nadia ElKhatib, Margot Fournier, Ahmed Dahmani, Elodie Montaudon, Guillaume Montagnac, Elisabetta Marangoni, and Philippe Chavrier**

Supporting Information

mTOR repression in response to amino acid starvation promotes ECM degradation through MT1-MMP endocytosis arrest

Cecilia Colombero, David Remy, Sandra Antoine-Bally, Anne-Sophie Macé, Pedro Monteiro, Nadia ElKhatib, Margot Fournier, Ahmed Dahmani, Elodie Montaudon, Guillaume Montagnac, Elisabetta Marangoni, and Philippe Chavrier[*]

Supplementary Experimental Section

Cell culture, transfection and siRNA treatment. Human MDA-MB-231 breast adenocarcinoma cells obtained from ATCC (ATCC HTB-26) were grown in L-15 medium (Sigma-Aldrich) supplemented with 15% fetal calf serum (FCS) and 2 mM Gln (ThermoFisher Scientific) at 37°C in 1% CO_2. The human pancreas adenocarcinoma cell line Bx-PC3 obtained from ATCC (ATCC CRL-1687) was grown in RPMI-1640 medium (ThermoFisher Scientific) supplemented with 10% fetal calf serum at 37°C in 5% CO_2. Both cell lines were routinely tested for mycoplasma contamination. MDA-MB-231 cells stably expressing $TKS5^{GFP}$ or $MT1\text{-}MMP^{pHLuorin}$ were generated by lentiviral transduction.[1] For transient expression, MDA-MB-231 cells were transfected with the plasmid constructs using AMAXA nucleofection (Lonza) and analyzed by live cell imaging 48 h after transfection. For starvation experiments, cells were cultured in EBSS medium (ThermoFisher Scientific) supplemented with MEM Vitamins (Gibco, composition in Table S1, Supporting Information) at 37°C in 5% CO_2. For siRNA treatment, except for α-adaptin and clathrin heavy chain (CHC), we used SMARTpool reagents consisting of a mix of four individual siRNAs used at lower concentration in order to reduce potential off-target effects (see Table S2, Supporting Information, for a list of siRNAs used for this study). Cells were treated with the indicated SMARTpool mix (50 nM final concentration) using Lullaby (OZ Biosciences) according to manufacturer instructions and analyzed after 72 hrs of treatment. For silencing of α-adaptin and CHC, cells were treated twice with the siRNA (50 nM final concentration) at 48 hrs interval and analyzed 120 hrs after initial treatment.

Antibodies and drugs. The source of commercial antibodies used for this study are listed in Table S3 (Supporting Information). The source and working concentration of drugs used in this study are listed in Table S4 (Supporting Information).

Polymerization of type I collagen gel. A type I collagen polymerization mix was prepared on ice by adding 25 µM HEPES (final concentration) to a 2.2 mg mL^{-1} acidic-extracted type I collagen solution (Corning) and pH was adjusted to 7.5 with 0.34 N NaOH. When required for microscopic visualization of the collagen network, 2 to 5% of a ~2 mg mL^{-1} solution of AlexaFluor 647-conjugated type I collagen was added to unlabeled collagen in the polymerization mix. When required, drugs were added to the appropriate final concentration in the polymerization mix (see Table S4, Supporting Information). Polymerization was started by incubation at 37°C in a humidified chamber (CO2 cell incubator).

Co-immunoprecipitation of MT1-MMP^{phLuorin}-bound proteins. Cells stably expressing MT1-MMP^{pHLuorin} from two 100-mm dishes (3×10^{6} cells per dish plated the day before) were pulled and lysed in 500 µl of lysis buffer (50 mM Tris-HCl, pH 7.5, 150 mM NaCl, 0.5 mM EDTA, 10 mM $MgCl_2$, 10% glycerol, 60 mM β-glucoside, 1% NP-40, Protease inhibitor cocktail tablets (Roche #11873580001) and phosphatase inhibition cocktail 2 (Sigma Aldrich #P5726) for 15 min at 4°C. Lysates were centrifuged at 17,700 x g for 10 min at 4°C. 50 µl of the supernant was kept as input and the rest was added to 30 µl of equilibrated control magnetic agarose beads (ChromoTek #bmab20) for 30 min at 4°C under mild rotation. The precleared lysate was then incubated with 30 µl of magnetic agarose beads coupled to anti-GFP nanobodies (GFPTrap; ChromoTek #gtma20) for 1 hr at 4°C under mild rotation. The beads were washed with washing buffer A (lysis buffer without β-glucoside and with 0.7% NP-40), followed by two washes with washing buffer B (without β-glucoside and

NP-40). Proteins bound to the beads were immediately heated at 95°C for 10 min in Laemmli Sample Reducing buffer (63 mM Tris/HCl, pH 6.8, 2% sodium dodecyl sulfate (SDS), 5% glycerol, 2% β-mercaptoethanol, 0.005% bromophenol blue) and stored at −20°C.

Western blot analysis. Cells treated under the indicated conditions were lysed in lysis buffer containing 50 mM Tris HCl (pH 8.0), 137 mM NaCl, 1% triton X-100, 10 mM MgCl2, 10% glycerol, Protease inhibitor cocktail tablets (Roche #11873580001) and phosphatase inhibition cocktail 2 (Sigma Aldrich #P5726). Lysates were centrifuged at maximum speed (17,700 x g) for 30 min at 4°C. 4x Laemmli Sample Reducing buffer was added and samples were heated for 10 min at 95°C and analyzed by SDS-polyacrylamide gel electrophoresis (PAGE) on NUPAGE 3-8% Tris-acetate or 4-12% Tris-glycine gels (ThermoFisher Scientific). Proteins were transferred on a nitrocellulose membrane using the iBlot2 Dry Blotting System (Invitrogen). After incubating the membranes in 5% BSA or 5% skimmed milk in TBS (Interchim #UPU75132)-Tween 1%, proteins were detected by immunoblotting analysis with the indicated antibodies (see Table S3, Supporting Information). Antibodies were detected using the Enhanced Chemiluminescence reagent (ECL, Amersham RPN2232) on the ChemiDoc MP Imaging System (Bio-Rad).

Quantification of pericellular collagenolysis. To measure pericellular collagenolysis on a thin layer of type I collagen gel, a 18-mm diameter glass coverslip was layered with 200 µl of the ice-cold 2.2 mg mL^{-1} AlexaFluor 647(AF^{647})-labeled type I collagen polymerization mix as described above. Excess collagen solution was removed by pipette aspiration to leave a thin smear of collagen solution on the glass coverslip. After 3 min of polymerization at 37°C, the collagen gel was gently washed in PBS and $7x10^4$ cells were added and incubated for 1 at 37°C in CM or EBSS

medium in the presence or in the absence of AA supplements or drugs as indicated. Cells were pre-extracted with 0.1% Triton X-100 in 4% PFA in PBS for 90 sec at 37°C and fixed in 4% PFA in PBS for 20 min at 37°C. Coverslips were treated with 1% BSA in PBS for 30 min at room temperature then incubated with Col1-¾C and anti-cortactin antibodies diluted in 1% BSA in PBS for 2 hrs at 4°C. After three washes with PBS at 4°C, samples were counterstained with Cy3-conjugated anti-rabbit IgG and A488-conjugated anti-mouse IgG antibodies for 60 min at 4°C, extensively washed in PBS and mounted in Prolong-DAPI mounting medium (Invitrogen). Images were acquired with a wide-field microscope (Eclipse 90i Upright; Nikon) using a 100x Plan Apo VC 1.4 oil objective and a cooled interlined charge-coupled device (CCD) camera (CoolSnap HQ^2; Roper Scientific). A z-dimension series of images was taken every 0.2 µm by means of a piezoelectric motor (Physik Instrumente). The system was steered by Metamorph software. Deconvolution was processed by Nikon NIS-Elements software (3D-deconvolution module; Lucy-Richardson algorithm).

For quantification of pericellular collagenolysis in a 3D collagen network, 40 µl of a $6x10^4$ cells/mL cell suspension in the 2.2 mg mL^{-1} type I collagen polymerization mix was added on top of a 12-mm diameter glass coverslip and polymerization was performed for 30 minutes at 37°C. The indicated culture medium was added and samples were incubated for 6 hrs at 37°C. Samples were fixed, permeabilized and stained with Col1-¾C antibody as described above except that samples were counterstained with Phalloidin-Alexa488 to visualize cell shape. Image acquisition was performed with an A1R Nikon confocal microscope with a 40x NA 1.3 oil objective using high 455 sensitivity GaASP PMT detector and a 595 +/- 50 nm band-pass filter. Quantification of Col1-¾C signal (cleaved collagen) was performed with a

homemade ImageJ macro. Acquired z-planes were projected using maximal intensity projection in Fiji and Col1-¾C signal was determined using the thresholding command excluding regions <50-px to avoid non-specific signal. Col1-¾C signal area was normalized to the total cell surface (thin layer) or to the number of nuclei in field (3D network) and values normalized to control cells.

Fluorescent gelatin degradation assay. MDA-MB-231 cells were plated for 1 to 5 hrs on Oregon Green 488 (OR^{488}) or AF^{594}-conjugated cross-linked gelatin (Invitrogen) in EBSS or CM medium in the presence or absence of rapamycin as previously described.[2] Cells were pre-extracted with 0.1% Triton X-100 in 4% PFA in PBS for 90 sec at 37°C and fixed in 4% PFA in PBS for 20 min at 37°C and then stained with the indicated antibodies or with fluorescently-labeled phalloidin to stain F-actin. Cells were imaged with a 100x objective on a wide-field microscope equipped with a piezoelectric motor as above. For quantification of degradation, the area of degraded matrix (black pixels) measured with the threshold command of ImageJ was divided by the total cell surface and values were normalized to control cells. The regions of interest delimiting the gelatin degradation were saved for further analysis, such as the assessment of AP-2 association (see below). Linescans were performed using Fiji software. Deconvolution was processed by Nikon NIS-Elements software (3D-deconvolution module; Lucy-Richardson algorithm).

Quantification of invadopodia parameters. 7x10^4 MDA-MB-231 cells stably expressing TKS5-GFP were plated on top of a coverslip coated with AF^{405}- or AF^{594}-conjugated gelatin and incubated for 1 hr in CM or EBSS medium. After fixation and permeabilization, cells were stained with anti-GFP antibodies. TKS5 positive structures were detected using the threshold command of ImageJ set with constant upper and lower threshold values. TKS5-positive structures outside of the regions of

degraded gelatin were eliminated from the analysis. The distance of the invadopodia to the cell centroid was measured on ImageJ with a homemade macro. Briefly, a line was created between the cell centroid and the invadopodia, then extended to the nearest cell periphery point. The reported distance is the distance between the invadopodia and the cell centroid normalized by the distance between the cell periphery and the cell centroid.

Quantification of CCP density. $7x10^4$ MDA-MB-231 cells were plated on top of a coverslip coated with gelatin^{OR488} and incubated for 1 hr in CM or EBSS medium. After fixation and permeabilization, cells were stained with α-adaptin as described above. CCPs in the entire cell were detected using the Find Maxima command of ImageJ and the number of detected CCPs was divided by the area of the cell. CCPs positions were saved for further analysis (see below).

Randomization of AP-2 distribution over gelatin degradation spots. To measure the association of α-adaptin positive CCPs with gelatin degradation spots, CCPs and degradation spots were detected as described above and their positions as well as the position of all pixels inside the cell (total pixels) defined by their X and Y coordinates were saved. For each CCP, (X, Y) positions were randomly drawn from all pixels of the cell, effectively changing the position of CCPs inside the cell in a random fashion (see Figure S3B, Supporting Information). This randomization procedure was performed 5,000 times per cell and the number of CCPs associated with gelatin degradation was measured each time. The true value of CCP association with gelatin degradation was calculated and compared to the randomized values. Synthetic images displaying cell contour (white line), degradation spots (black) and associated CCPs (red crosses) were generated with ImageJ. This procedure was

repeated for ten independent cells with *p*-values ranging from 0 to 0.0142 (mean *p*-value = 0.001).

Tfn uptake assay. 7x10^{4} MDA-MB-231 cells plated on a 18-mm diameter glass coverslip were incubated overnight at 37°C in CM. Cells were washed twice with PBS before incubation in EBSS or CM medium for 1 hr at 37°C, then transferred on ice and washed twice with ice-cold EBSS or L15 medium supplemented with 1% BSA and 20 mM HEPES pH 7.5. Coverslips were incubated with 20 µg mL^{-1} of AF^{546}-conjugated Tfn (ThermoFisher) in the same medium for 1 hr at 4°C. Cells were fixed with 4% PFA in PBS or incubated in pre-heated CM or EBSS for 2, 5 or 10 min at 37°C before fixation. After permeabilization with 0.1% Triton X-100 in PBS for 15 min, samples were incubated with anti-α-adaptin (overnight at 4°C) or with anti-EEA1 antibodies (1 hr at room temperature), and then counterstained with AF488-conjugated anti-mouse antibodies (1 hr at room temperature). Stacks of images were acquired with a wide-field microscope (Eclipse 90i Upright; Nikon) steered by Metamorph software as described above. For analysis, the plane corresponding to the plasma membrane was selected. CCPs positive for α-adaptin in a selected region were detected and segmented using the manual threshold command of ImageJ. The regions of interest (ROI) were saved and copied on the Tfn image. The mean intensity of Tfn inside each ROI was measured and a frequency histogram was generated with a normalization to T0.

Quantification of LC3-positive puncta. 7x10^{4} MDA-MB-231cells were plated on collagen-coated or on non-coated 18-mm diameter glass coverslips as previously described and incubated for 4 at 37°C in CM or in EBSS medium. Cells were fixed with 4% PFA in PBS for 10 min and permeabilized with 0.05% saponin (Sigma-Aldrich) in PBS for 10 min. Samples were blocked in PBS with 0.05% saponin and

5% FCS for 30 min at room temperature and stained with anti-LC3 and anti-p4E-BP1 antibodies for 2 hrs at room temperature. After three washes, samples were counterstained with Cy3-conjugated anti-mouse IgG and Alexa488-conjugated anti-rabbit IgG antibodies and mounted in Prolong-DAPI medium. Image acquisition was performed by wide-field microscopy as previously described. Quantification of LC3-positive vesicles was performed by maximal orthogonal projection of the series of optical sections (the distance between two sections is 0.2 µm). Cells were manually delimited using the p4E-BP1 signal while LC3 signal was denoised and thresholded to detect LC3-positive autophagic vesicles. Detected spots were counted and saved for visual verification. No manual correction was done. The average number of LC3-positive puncta per cell was normalized to the value in CM-treated cells set to 1.

Dynamics of TKS5- and µ-adaptin-positive structures by live cell total internal reflection fluorescence microscopy (TIRF-M). MDA-MB-231 cells transfected with GFP-tagged TKS5 and mCherry-tagged µ-adaptin were plated in CM or EBSS on glass bottom dishes (Ibidi Corporation) layered with unlabeled cross-linked gelatin as previously described. Simultaneous dual color TIRF-M sequences were acquired with an inverted microscope (Eclipse-Ti-E, Nikon) equipped with a 100x PlanApo TIRF objective (1.47 NA), a TIRF arm, an image splitter (DV; Roper Scientific) installed in front of the EMCCD camera (Photometrics) and a temperature controller. GFP and m-Cherry were excited with 491- and 561-nm lasers, respectively (50 mW, Gataca Systems) and fluorescent emissions were selected with bandpass and longpass filters (Chroma Technology Corp). The system was driven by Metamorph. For quantification of CCP dynamics, CCP lifetime was measured using the TrackMate plugin of FIJI.[3] At least 300 CCPs from at least 6 cells per condition and per

experiment were tracked from three independent experiments. Data are expressed as mean lifetime ± sem.

***Ex-vivo* culture of TNBC patient-derived xenografts.** Breast cancer patient derived xenografts were obtained from triple-negative breast tumors and generated as described.[4] After surgical excision of the tumor xenograft, tumor cells were dissociated in DMEM/F12 medium supplemented with collagenase and hyaluronidase (SIGMA-Aldrich, 1X final) in 10 mM HEPES, 7.5% BSA Fraction V (Gibco), 5 µg mL^{-1} insulin (SIGMA-Aldrich) and 50 µg mL^{-1} gentamycin (GIBCO) for 1 hr at 37°C on a rotating wheel at 180 rpm as previously described.[5] Samples were washed with DMEM/F12 medium and digested with 0.25% of trypsin (Gibco) for 2 min at 37°C. Trypsin was neutralized in HBSS medium (Invitrogen) supplemented in 10 mM HEPES and 2% FCS. Then, samples were treated with dispase (5 UI mL^{-1}, StemCell Technologies) and DNAse I (1mg mL^{-1} in DMEM, Sigma for 2 min at room temperature and then incubated in neutralization buffer supplemented with NH_4Cl (0.8%, StemCell Technologies) to remove red blood cells. After filtration through a 40 µm Cell Strainer (Corning), tumor cells were plated in a 25-cm^2 cell-culture flask for 16 hrs at 37°C in DMEM/F12 medium supplemented with 10% FCS. For the pericellular collagenolysis assay, non-attached PDX tumor cells in the culture supernatant were resuspended in a 2,2 mg mL^{-1} collagen I solution as described above and incubated for 16 hrs in CM or EBSS medium with or without GM6001. After fixation with PFA 4% for 20 min and permeabilization with Triton 0.1% in PBS for 5 min, samples were stained with Col1-¾C and anti-Keratin-8 (K8) antibodies (2 hrs at 4°C) or anti-phospho-4E-BP1 and anti-Keratin-8 antibodies (1 hr at 4°C), counterstained with fluorescently labeled secondary antibodies and mounted. Image

acquisition was performed with an A1R Nikon confocal microscope as described above.

Statistics and reproducibility. All data are presented as mean ± S.E.M. from at least three independent experiments except indicated otherwise. GraphPad Prism software was used for statistical analysis. Data were tested for normal distribution using the D'Agostino-Pearson normality test and nonparametric tests were applied otherwise. One-way ANOVA, Kruskal-Wallis or Mann-Whitney tests were applied as indicated in the figure legends and are summarized in Supplemental Table 5. Statistical significance was defined as *, $P<0.05$; **, $P<0.01$; ***, $P<0.001$; ****, $P<0.00001$; ns, not significant.

Supplementary Figure legends

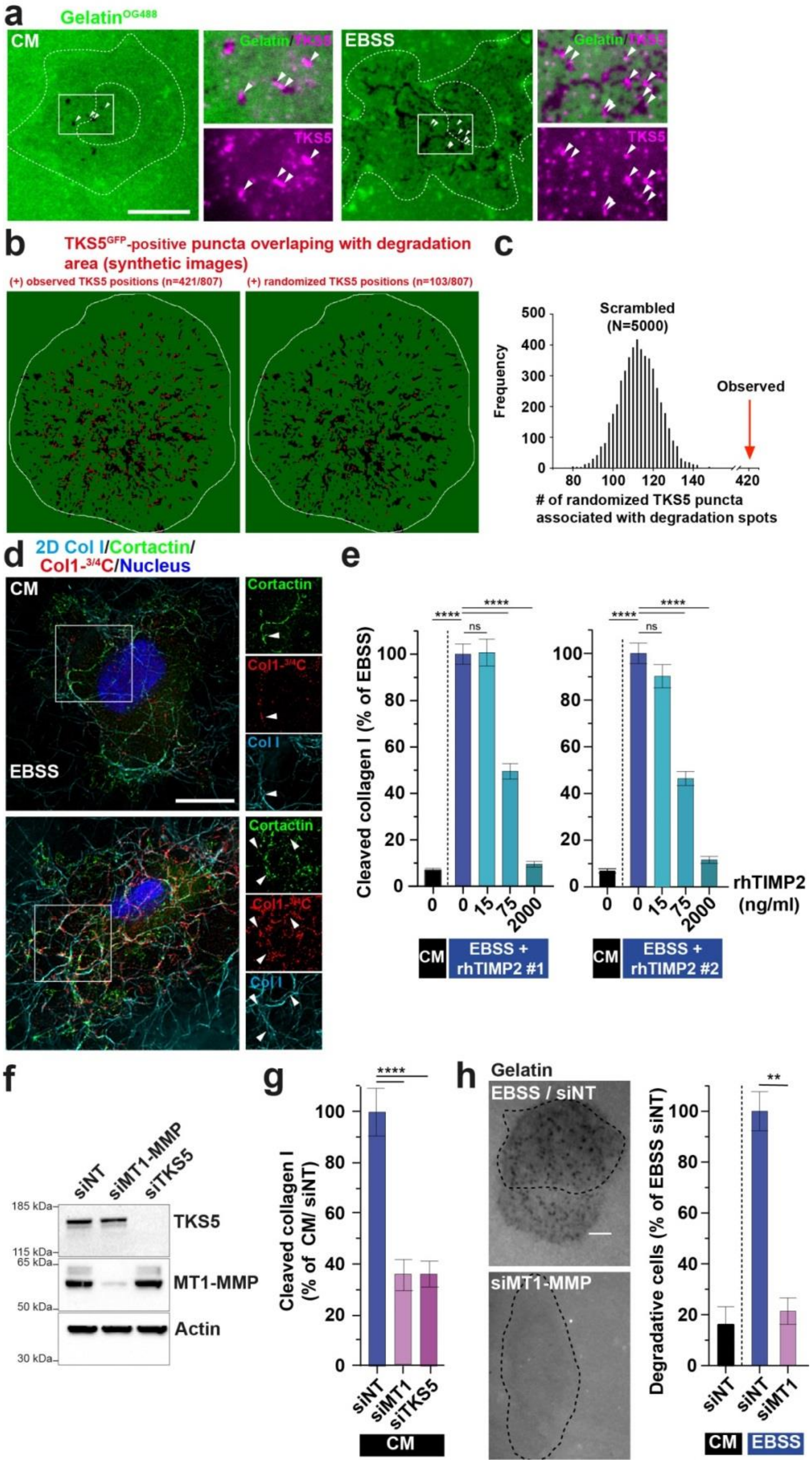

Figure S1. Matrix degradation by starved cells. (a) MDA-MB-231 cells plated on fluorescently-labeled gelatin (green) for 60 min in CM or EBSS starvation medium depleted for AAs and serum and stained for TKS5 (magenta). Higher magnification of

boxed regions is shown in the insets. Arrowheads, TKS5-positive invadopodia; dotted lines, cell and nucleus contour. Scale bar, 10 µm. **(b)** Synthetic images showing virtual TKS5-positive invadopodia (depicted as red crosses) overlapping with the mask representing the degraded zones of gelatin (black spots over a green background). In the left image, the position of TKS5-positive structures corresponds to their observed position in the original microscopy image (see Figure 1A, EBSS). The right image corresponds to one of the 5,000 scrambled images generated by randomization of CCP positions on the mask of the degradation zones. **(c)** $TKS5^{+}$ invadopodia were scrambled 5,000 times and the histogram shows the number of randomized $TKS5^{+}$ invadopodia associated with gelatin degradation spots. The true unscrambled value (n=422) exceeds the randomized values, indicating high statistical confidence in non-random association of $TKS5^{+}$ invadopodia with degradation areas (see also Supplemental Table 5). **(d)** Deconvoluted images showing MDA-MB-231 cells cultured on a fibrillar type I collagen network (cyan) for 60 min in indicated medium and stained for cortactin (green); cleaved collagen fibers (red); nucleus (blue). Scale bar, 10 µm. Higher magnification of boxed regions is shown in the insets. Arrowheads, cortactin-positive invadopodia. **(e)** Collagen cleavage by MDA-MB-231 cells incubated for 60 min in CM or EBSS medium supplemented with 15, 75 or 2000 ng mL^{-1} recombinant human TIMP2 protein from two independent suppliers (rhTIMP2#1 and #2). **(f)** Representative western blots of MT1-MMP and TKS5 expression with actin as loading control in MDA-MB-231 cells treated with indicated siRNAs. **(g)** Collagen cleavage by MDA-MB-231 cells knocked-down for MT1-MMP or TKS5 or treated with a non-targeting siRNA and cultured in CM. **(h)** MDA-MB-231 cells knocked-down for MT1-MMP or treated with a non-targeting (siNT) siRNA were plated on fluorescently-labeled gelatin in CM or EBSS

medium for 2 hrs. The cell contour is shown with a black dotted line. Scale bar, 10 µm. The graph shows the gelatin degradation normalized to the degradation of cells grown in EBSS medium ± SEM.

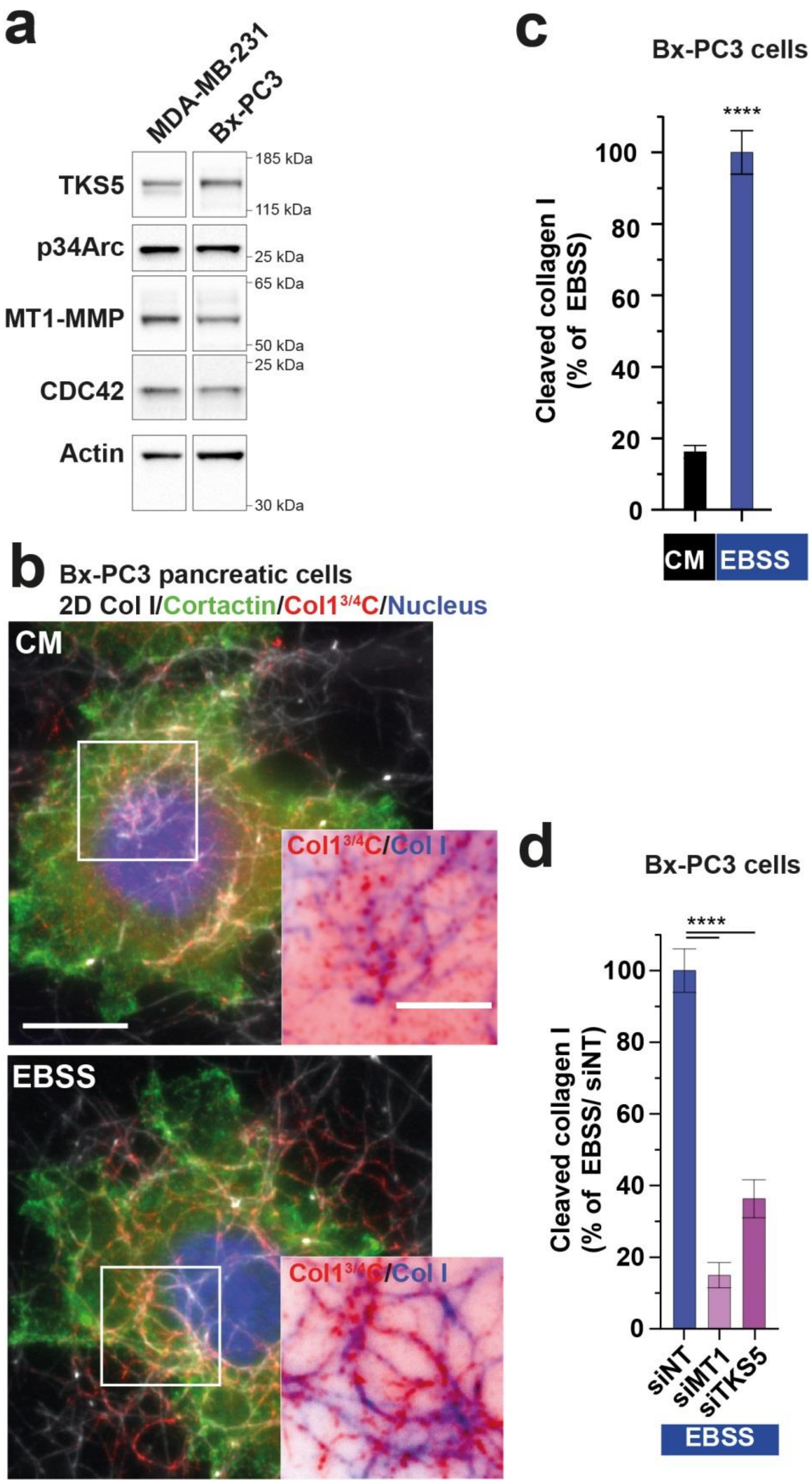

Figure S2. AA starvation induces matrix degradation in pancreatic Bx-PC3 tumor cells. (a) Comparison of the expression of key invadopodia components by immunoblotting analysis in MDA-MB-231 and Bx-PC3 cell lysates with actin as loading control. Molecular weights are in kDa. **(b)** Pancreatic ductal adenocarcinoma Bx-PC3 cells were cultured on a layer of fibrillar type I collagen (gray) for 60 min in indicated medium and stained for cortactin (green) and cleaved collagen I (Col1-¾C, red). Scale bar, 10 µm. Insets, higher magnification of boxed regions using inverted lookup tables (collagen fibers are in blue, cortactin or Col1-¾C signal is in red). Scale bar, 5 µm. **(c)** Collagen cleavage by Bx-PC3 cells was measured by Col1-¾C neoepitope staining and normalized to mean value of cells starved in EBSS ± SEM. **(d)** Collagen cleavage by Bx-PC3 cells knocked-down for MT1-MMP or TKS5 or treated with a non-targeting siRNA and cultured in EBSS medium.

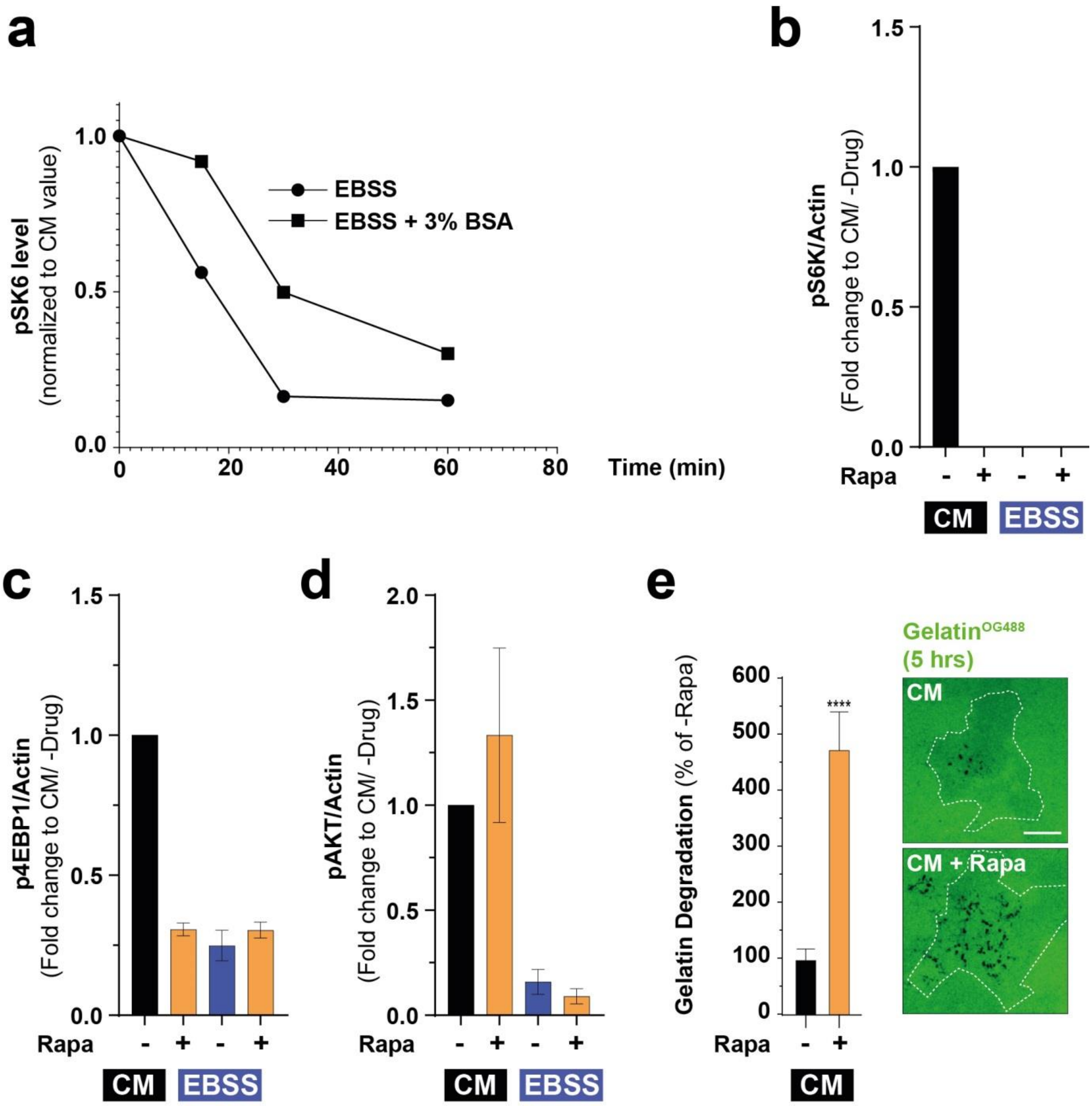

FIGURE S3

Figure S3. Phosphorylation of mTOR substrates in cells treated with rapamycin. (a) Levels of phosphorylated S6K in MDA-MB-231 cells cultured in EBSS medium in the absence or presence of 3% BSA normalized to pSK6 levels in CM medium from two independent experiments (see Figure 3C). **(b-d)** Levels of phosphorylated (p)S6K (panel A), p4E-BP1 (panel B) or pAKT (panel C) normalized to actin levels in MDA-MB-231 cells cultured in CM or EBSS medium in the presence

or absence of rapamycin from three independent experiments (see Figure 3E). **(e)** Gelatin degradation by MDA-MB-231 cells incubated for 5 hrs in CM with or without rapamycin. Scale bar, 10 µm.

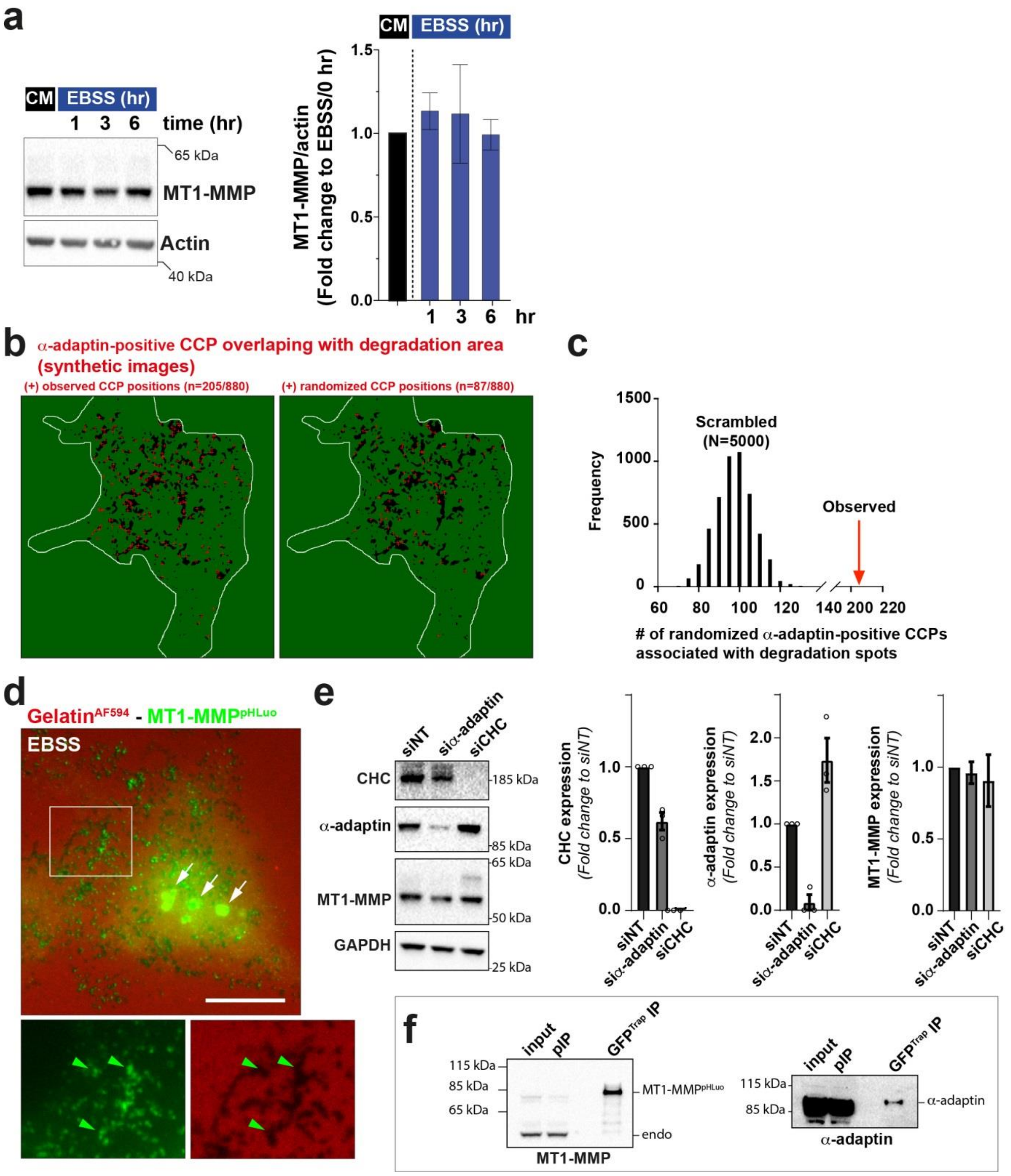

Figure S4. Matrix degradation correspond to MT1-MMP accumulation in arrested CCPs. (a) MT1-MMP levels analyzed by western blot, normalized to actin in MDA-MB-231 cells incubated in the indicated medium for the indicated periods of

time. Representative immunoblots are shown in the right panels. Molecular weight in kDa. **(b)** Synthetic images showing virtual CCPs (depicted as red crosses) overlapping with the mask representing the degraded zones of gelatin (black spots over a green background). In the left image, the position of CCPs corresponds to their observed position in the original microscopy image (see Figure 4D, EBSS). The right image corresponds to one of the 5,000 scrambled images generated by randomization of CCP positions. **(c)** TKS5$^+$ invadopodia were scrambled 5,000 times and the histogram shows the number of randomized TKS5$^+$ invadopodia associated with gelatin degradation spots. The true unscrambled value (n=422) exceeds the randomized values, indicating high statistical confidence in non-random association of TKS5$^+$ invadopodia with degradation areas (see also Supplemental Table 5). **(d)** MDA-MB-231 cells expressing MT1-MMP^{pHLuorin} were plated on AF^{594}-labeled gelatin for 60 min. White arrows, fluorescence signal of MT1-MMP^{pHLuorin} in endolysosomes. Green arrowheads point to the accumulation of MT1-MMP^{pHLuorin} in association with gelatin degradation areas. Scale bars, 10 µm. **(e)** Representative western blots of CHC, α-adaptin or MT1-MMP expression with GAPDH as loading control in MDA-MB-231 cells treated with indicated siRNAs. Molecular weights are in kDa. Quantification of protein expression based on three (α-adaptin and CHC) or two (MT1-MMP) independent experiments. **(f)** Lysates of MDA-MB-231 cells expressing MT1-MMP^{pHLuorin} were immunoprecipitated with GFP antibodies (GFP^{Trap} IP). Total lysate before (input) and after immunoprecipitation (pIP) was loaded as control. Bound proteins were analyzed with MT1-MMP and α-adaptin antibodies Equal loading was controlled using GAPDH antibody (not shown).

Supplementary Table Legends

Table S1. Medium composition

Vitamin mix

Component	MEM Vitamin Solution 100 X (g/L)	L-15 Medium (g/L)
**Choline Chloride**	0.1	0.001
**Folic Acid**	0.1	0.001
**Myo-Inositol**	0.2	0.002
**Niacinamide**	0.1	0.001
**D-Panthothenic Acid * ½Ca**	0.1	0.001
**Pirydoxal.HCl**	0.1	0.001
**Riboflavin**	0.01	0.0001
**Thiamine*HCl**	0.1	0.001

Amino acid mix composition

Amino Acid	RPMI-1640 50 X (g/L)	L-15 Medium (g/L)
**L-Alanine**	-	0.225
**L-Arginine (free base)**	10.0	0.5
**L-Asparagine**	2.84	0.25
**L-Aspartic Acid**	1.0	-
**L-Cystine**	2.5	0.12
**L-Glutamic Acid**	1.0	0.3
**Glycine**	0.5	0.2
**L-Histidine**	0.75	0.25
**Hydroxy-L-Proline**	1.0	-
**L-Isoleucine**	2.5	0.125
**L-Leucine**	2.5	0.125
**L-Lysine**	2.0	0.094
**L-Methionine**	0.75	0.075
**L-Phenylalanine**	0.75	0.125
**L-Proline**	1.0	-
**L-Serine**	1.5	0.2
**L-Threonine**	1.0	0.3
**L-Tryptophan**	0.25	0.02
**L-Tyrosine**	1.16	0.3
**L-Valine**	1.0	0.1

Table S2. siRNAs used in this study

siRNA	Company	Targeted sequence (5'-to-3')
**MT1-MMP** *SMARTpool*	Horizon Discovery L-004145-00-0005	GGAUGGACACGGAGAAUUU<br>GGAAACAAGUACUACCGUU<br>GGUCUCAAAUGGCAACAUA<br>GAUCAAGGCCAAUGUUCGA
**TKS5** *SMARTpool*	Horizon Discovery L-006657-00-0005	ACAAUAACCUCAAAGAUGU<br>GGACGUAGCUGUGAAGAGA<br>CGACGGAACUCCUCCUUUA<br>GGAUAAGUUUCCCAUUGAA
**α-adaptin**	Merck Millipore	AAGAGCAUGUGCACGCUGGCCA
**Clathrin Heavy Chain (CHC)**	Elkhatib et al., Science 2017	GCUGGGAAAACUCUUCAGATT
**Non-Targeting**	Horizon Discovery D-001810-01	UGGUUUACAUGUCGUACUAA
**siTSC2** *SMARTpool*	Horizon Discovery L-003029-00-0005	GCAUUAAUCUCUUACCAUA<br>CGAACGAGGUGGUGUCCUA<br>GGAAUGUGGCCUCAACAAU<br>GGAUUACCCUUCCAACGAA
**siTBC1D7** *SMARTpool*	Horizon Discovery L-021140-00-0005	GGAAGAUAGUGUCGACUGU<br>UUACAGAGGGUUUGGGAUA<br>CCAUUAAAUACCAAGUAC<br>CGCCCAAACUUCCUUAUGA

Table S3. Commercial antibodies and immunolabeling reagents used in this study.

Antigen	Company	Type (species)
**Collagen type I cleavage site (Col1–¾C)**<br>*IF*	ImmunoGlobe (0217-050)	Polyclonal (Rabbit)
**Cortactin**<br>*IF*	Merck (clone 4F11, 05-180)	Monoclonal (mouse)
**MT1-MMP (MMP14)**<br>*IF, WB*	Merck (clone LEM-2/15.8, MAB3328)	Monoclonal (mouse)
**TKS5 (SH3PXD2A)**<br>*IF, WB*	Novus Biologicals (NBP1-90454)	Polyclonal (rabbit)
**Paxillin**<br>*IF*	BD Transduction Laboratories (610052)	Monoclonal (mouse)
**GFP**<br>*IF*	Abcam (ab13970)	Polyclonal (chicken)
**mTOR**<br>*IF*	Cell Signaling TECHNOLOGY (2983S)	Monoclonal (rabbit)
**TSC2**<br>*WB*	Cell Signaling TECHNOLOGY (4308S)	Monoclonal (rabbit)
**TBC1D7**<br>*WB*	Cell Signaling TECHNOLOGY (1494S)	Monoclonal (rabbit)
**LC3**<br>*IF*	MBL (clone 4E12 M152-3)	Monoclonal (mouse)
**LC3**<br>*WB*	Cell Signaling TECHNOLOGY (2775S)	Polyclonal (rabbit)
**Alpha adaptin 2 (AP2)**<br>*IF*	Abcam (ab2730)	Monoclonal (mouse)
**Alpha adaptin 2 (AP2)**<br>*WB*	Abcam (ab2807)	Monoclonal (mouse)
**Early Endosome Antigen 1 (EEA1)**	BD Transduction Laboratories (610457)	Monoclonal (mouse)
**Phospho-4E-BP1 (Ser65)**<br>*IF, WB*	Cell Signaling TECHNOLOGY (clone D9G1Q 13443S)	Polyclonal (rabbit)
**Phospho-(p70)S6 Kinase (Thr389)**<br>*WB*	Cell Signaling TECHNOLOGY (9205)	Polyclonal (rabbit)
**Phospho-AKT (Ser473)**<br>*WB*	Cell Signaling TECHNOLOGY (4060)	Polyclonal (rabbit)
**(p70)S6 Kinase**<br>*WB*	Cell Signaling TECHNOLOGY (clone 49D7 2708)	Monoclonal (rabbit)
**AKT**<br>*WB*	Cell Signaling TECHNOLOGY (9272)	Polyclonal (rabbit)
**β1 integrin**<br>*WB*	Gift from C. Albiges-Rozo	Polyclonal (rabbit)
**Cytokeratin-8**<br>*IF*	DSHB	Monoclonal (rat)
**Actin**<br>*WB*	Sigma-Aldrich (clone AC-15 A1978)	Monoclonal (mouse)
**GAPDH**<br>*WB*	Santa Cruz Biotechnology (25788)	Polyclonal (rabbit)

**Alpha tubulin**<br>*WB*	Sigma-Aldrich (T-9026)	Monoclonal (mouse)
**HRP-conjugated anti-rabbit IgG**	Sigma (A0545)	Goat
**HRP-conjugated anti-mouse IgG**	Jackson ImmunoResearch (115-035-062)	Goat
**Alexa Fluor 488 Phalloidin**	Molecular Probes (A12379)	
**Anti-rabbit Alexa488**	Molecular Probes (A21206)	Goat
**Anti-rabbit Cy3**	Molecular Probes (A21206)	Donkey
**Anti-chickenAlexaFluor488**	Molecular Probes (A11039)	Donkey
**Anti-mouse Cy3**	Jackson ImmunoResearch (715-165-151)	Donkey
**Anti-rat Alexa488**	Molecular Probes (A21208)	Donkey

Table S4. Chemicals and reagents used in this study

Reagent	Company	Reference	Vehicle	Dilution
**RPMI 1640 amino acids solution**	Sigma-Aldrich	R7131	Medium	1/100
**Bovine serum albumin solution 30%**	ThermoFischer Scientific	A7284	Medium	3%
**GM6001**	Merck Millipore	CC1100	Ethanol	40 µM
**Rapamycin**	Tocris Biotechne	1292	Ethanol	20 nM
**Recombinant human tissue inhibitor of metalloproteinase (rhTIMP-2)**	- R&D Systems (rhTIMP-2#1)<br>- Sigma-Aldrich (rhTIMP-2#2)	971-TM-010<br><br>SRP3174	Medium	15, 75 or 2000 ng/mL
**Transferrin from human serum, Alexa Fluor™ 546 Conjugate**	Invitrogen	11530766	Medium	20 µg/ml

Table S5. Analyzed variables and statistics used in this study.

Table S5. Analyzed variables and statistics used in this study.

Figure	Conditions	Mean	SEM	n	N	p value	Test
1-b **Gelatin degradation** *(fold change to CM)*	**CM**	**1**	**0.2**	**42**	2	**-**	K-W
	EBSS 30 min	4.2	0.4	42		<0.00001	
	EBSS 60 min	8.5	0.8	53		<0.00001	
	EBSS 120 min	14.2	1.1	42		<0.00001	
	EBSS + GM6001 60 min	2.9	0.6	42		ns	
1-c **Density of TKS5+ invadopodia** *(per μm^2)*	**CM**	**0,03**	**0,003**	**51**	3	**-**	M-W
	EBSS	0,15	0,011	54		<0.00001	
1-d **Average size of TKS5+ invadopodia** *(μm^2)*	**CM**	**0,23**	**0,022**	**51**	3	**-**	M-W
	EBSS	0,11	0,004	54		<0.00001	
1-e **Distance of TKS+ invadopodia to the centroid** *(µm)*	**CM**	**0,408**	**0,006**	**1172**	3	**-**	M-W
	EBSS	0,544	0,003	6850		<0.00001	
1-g **Cleaved collagen I** *(% of EBSS)*	CM	35.7	3.5	59	3	<0.00001	M-W
	**EBSS**	**100**	**6.2**	**54**		**-**	
1-i **Cleaved collagen I** *(% of EBSS/siNT)*	CM siNT	16.1	1.0	57	3	<0.00001	K-W
	**EBSS siNT**	**100**	**5.9**	**63**		**-**	
	EBSS siMT1	10.6	1.4	62		<0.00001	
	EBSS siTKS5	17.0	2.5	59		<0.00001	
3-b **Cleaved collagen I** *(% of EBSS)*	CM	16.3	1.1	87	4	<0.0001	K-W
	**EBSS**	**100**	**4.1**	**90**		**-**	
	EBSS + AA	53.8	3.1	93		<0.0001	
	EBSS + AA + Rapa	97.0	3.9	98		ns	
3-d **Cleaved collagen I** *(% of EBSS/-BSA)*	CM	2.6	0.4	91	3	<0.00001	K-W
	**EBSS**	**100**	**4.8**	**95**		**-**	
	EBSS+BSA	41.5	3.3	95		<0.00001	
3-f **Cleaved collagen I**	**CM**	**100**	**8.0**	**90**	4	**-**	M-W

(% of - Rapa)	CM + Rapa	269.4	39.6	92		<0.00001	
	**EBSS**	**100**	**4.0**	**64**	3	**-**	M-W
	EBSS + Rapa	111.8	3.4	67		0.0065	
3-i **Gelatin degradation (% of siNT)**	**EBSS siNT**	**100**	**3.2**	**68**	3	-	K-W
	EBSS siTSC2	48.5	2.3	56		<0.0001	
	EBSS siTSC2 + siTBC1D7	52.5	3.5	44		<0.0001	
3-k **LC3 puncta per cell** *(Fold change to CM)*	**CM - CollagenI**	**1.0**	**0.1**	**78**	3	**-**	K-W
	EBSS 4hrs - CollagenI	2.0	0.2	102		<0.00001	
	EBSS 4hrs +CollagenI	0.8	0.1	87		ns	
	EBSS 4hrs +CollagenI +GM6001	1.6	0.1	88		NA	
	EBSS 7hrs - CollagenI	2.1	0.2	104		<0.00001	
	EBSS 7hrs +CollagenI	0.8	0.1	101		ns	
	EBSS 7hrs +CollagenI +GM6001	1.4	0.1	94		NA	
4-c **alpha-adaptin+ CCP density** *(CCP/μm^2)*	CM	0.52	0.02	41	4	-	M-W
	EBSS	0.8	0.02	60		<0.0001	
4-f **% of AP2-positive CCPs (CM)**	T0	100	NA	4459	3	NA	NA
	T2	120		2955			
	T5	80		1923			
	T10	80		1925			
4-F **% of AP2-positive CCPs (EBSS)**	T0	100	NA	5827	3	NA	NA
	T2	100		3614			
	T5	100		3776			
	T10	120		2121			
4-h **Stable CCPs** *(% of total)*	CM	10.2	0.2	607	2	NA	NA
	EBSS	19.9	1.3	365			
4-j **Gelatin Degradation** *(% of EBSS siNT)*	**EBSS siNT**	**100**	**5.0**	**54**	3	**-**	K-W
	EBSS siα-adaptin	17.0	4.1	50		<0.0001	
	EBSS-siCHC	85.6	5.1	48		n.s.	

Supplementary Figure	Cell	Mode of randomized values	Min-Max of randomized values	True value	p-value
S1-b **Randomization of TKS5 positions**	1	225	195-264	323/733	0
	2	174	140-214	308/804	0
	3	445	64-120	218/468	0
	4	555	495-630	670/435	0
	5	243	201-288	518/824	0
	6	158	124-200	349/1038	0
	7	94	68-124	335/886	0
	8	46	28-64	160/325	0
	9	112	80-142	421/807	0
	10	68	44-88	210/605	0

Supplementary Figure	Condition	Mean	SEM	n	N	p value	Stat.
S1-e **Cleaved collagen I** *(% of EBSS/-rhTIMP2)* Left	CM	6.9	0.9	46	3	<0.00001	K-W
	**EBSS**	**100**	**4.4**	**85**		**-**	
	EBSS+15 ng/mL	100.6	5.8	82		ns	
	EBSS+75 ng/mL	49.5	3.3	80		<0.00001	
	EBSS+2000 ng/mL	9.5	1.2	77		<0.00001	
S1-e **Cleaved collagen I** *(% of EBSS/-rhTIMP2)* Right	CM	6.9	0.9	46	2	<0.00001	K-W
	**EBSS**	**100**	**4.4**	**85**		**-**	
	EBSS+15 ng/mL	90.2	5.0	49		ns	
	EBSS+75 ng/mL	46.4	3.0	52		<0.00001	
	EBSS+2000 ng/mL	11.6	1.5	41		<0.00001	
S1-g **Cleaved collagen I** *(% of CM/ siNT)*	**CM siNT**	**100**	**9.4**	**57**	3	**-**	K-W
	CM siMT1	35.8	6.2	43		<0.00001	
	CM siTKS5	36.2	4.9	45		<0.00001	
S1-h **Degradative cells** *(% of EBSS/ siNT)*	CM siNT	16.1	1.5	43	3	NA	M-W
	**EBSS siNT**	**100**	**5.9**	**63**		**-**	
	EBSS siMT1	10.6	1.4	62		0.0015	
S2-c **Cleaved collagen I** *(% of EBSS)*	CM	16.3	1.8	67	3	<0.00001	M-W
	**EBSS**	**100**	**6.1**	**88**		**-**	
S2-d **Cleaved collagen I** *(% of EBSS/ siNT)*	**EBSS siNT**	**100**	**6.0**	**96**	3	**-**	K-W
	EBSS siMT1	15.0	3.5	84		<0.00001	
	EBSS siTKS5	36.3	5.3	70		<0.00001	
S3-a **pS6K level**	EBSS T0	1.0	NA	NA	2	NA	NA
	EBSS T15	0.56					

(normalized to CM value)	EBSS T30	0.16					
	EBSS T60	0.15					
	EBSS + BSA T0	1.0					
	EBSS + BSA T15	0.92					
	EBSS + BSA T30	0.50					
	EBSS + BSA T60	0.30					
S3-b **pS6K/Actin** *(Fold change to CM/ -Drug)*	**CM**	**1**	**0**	NA	3	NA	NA
	Others	0	4.0				
S3-c **p4E-BP1/Actin** *(Fold change to CM/ -Drug)*	**CM**	**1**	**0**	NA	3	NA	NA
	CM + Rapa	0.3	0.02				
	EBSS	0.2	0.05				
	EBSS + Rapa	0.3	0.03				
S3-d **pAKT/Actin** *(Fold change to CM/ -Drug)*	**CM**	**1**	**0**	NA	3	NA	NA
	CM + Rapa	1.3	0.4				
	EBSS	0.2	0.06				
	EBSS + Rapa	0.1	0.04				
S3-e **Gelatin Degradation** *(fold change to CM)*	**CM**	**100.0**	**17.4**	**53**	2	**-**	M-W
	CM + Rapa	472.0	68.0	54		<0.0001	
S4-a **MT1-MMP/Actin** *(Fold change to EBSS/0 hr)*	**CM**	**1.0**	**0**	NA	2	**-**	K-W
	EBSS 1h	1.0	0.1			ns	
	EBSS 3hrs	0.8	0.3			ns	
	EBSS 6hrs	0.9	0.1			ns	

**Supplementary Figure**	**Cell**	**Mode of randomized values**	**Min-Max of randomized values**	**True value**	**p-value**
S4-c **Randomization of AP2 positions**	1	20	05-40	46/350	0
	2	41	16-63	91/641	0
	3	40	20-64	61/514	0
	4	50	27-74	127/442	0.0016
	5	40	20-65	54/394	0
	6	59	34-92	118/703	0.0142
	7	117	83-153	165/682	0
	8	98	63-133	204/880	0
	9	61	36-88	143/879	0
	10	51	30-77	112/575	0

**Supplementary Figure**	**Condition**	**Mean**	**SEM**	**n**	**N**	**p value**	**Stat.**
S4-e **CHC expression**	**siNT**	**1.0**	**0**	-	3	NA	NA
	siα-adaptin	0.6	0.06				

(Fold change to siNT)	siCHC	0	0				
S4-e**α-adaptin expression** *(Fold change to siNT)*	**siNT**	**1.0**	**0**	-	3	NA	NA
	siα-adaptin	0.1	0.1				
	siCHC	1.7	0.3				
S4-e **MT1-MMP expression** *(Fold change to siNT)*	**siNT**	**1.0**	**0**	-	2	NA	NA
	siα-adaptin	0.9	0.07				
	siCHC	0.9	0.2				

SEM (standard error of the mean); n, sample number; N, number of independent experiments; ns, not significative; NA, not available.

Data were tested for normal distribution using the D'Agostino-Pearson normality test and nonparametric tests were applied otherwise.

Non-parametric tests: K-W, Kruskal-Wallis test; M-W, Mann-Whitney

Parametric tests: One-Way ANOVA

Statistical significance was defined as *, P <0.05; **, P <0.01; ***, P <0.001; ****, P <0.00001; ns, not significant.

Supplementary Movie Legends

Movie S1: TKS5 localizes to highly dynamic matrix fiber-remodeling elongated invadopodia in cells grown in nutrient-replete conditions. Shown is a MDA-MB-231 cell expressing TKS5^{GFP} (green). The cell is plated on a layer of fibrillar collagen (magenta) and is grown in nutrient-replete conditions. Images were acquired every 1 min for 60 min. The last sequence of the movie are still images of the time projection of seven frames separated by a 10-min interval with the different time points represented with the indicated pseudocolor coding. In nutrient-proficient medium, cells form dynamic elongated TKS5-positive invadopodia in association with the underlying collagen fibers that are actively remodeled.

Movie S2: TKS5 localizes to punctate, mostly static, structures in starved cells. Shown is a MDA-MB-231 cell grown in nutrient-replete conditions and plated on a layer of fibrillar collagen (magenta). The cell expresses TKS5^{GFP} (green). Images were acquired every 1 min for 60 min. The last sequence of the movie is a time projection of seven frames separated by a 10-min interval with the different time points represented with the indicated pseudocolor coding. Starved cells form static TKS5-positive puncta with minimal displacement of the underlying matrix fibers.

Movie S3: CCP and TKS5 dynamics in nutrient replete and deplete conditions. Shown is the dynamics of plasma membrane TKS5^{GFP} (green) and CCPs labeled with μ-adaptin^{mCherry} (red) in MDA-MB-231 cells plated on cross-linked gelatin in CM (upper panel) or EBSS medium (lower panel). Images were acquired every 5 s for 5 min by TIRF-M. In nutrient-replete conditions (CM), large and static TKS5^{GFP}-positive

invadopodia form and TKS5 shows some transient association with CCPs, contrasting with long-lasting TKS5 interaction with CCPs observed in starved cells (yellow arrows). Scale bar, 2 µm.

Supplementary references

[1] R. Ferrari, G. Martin, O. Tagit, A. Guichard, A. Cambi, R. Voituriez, S. Vassilopoulos, P. Chavrier, *Nat Commun* **2019**, *10* (1), 4886, https://doi.org/10.1038/s41467-019-12930-y.

[2] M. Sakurai-Yageta, C. Recchi, G. Le Dez, J. B. Sibarita, L. Daviet, J. Camonis, C. D'Souza-Schorey, P. Chavrier, *J Cell Biol* **2008**, *181* (6), 985, https://doi.org/10.1083/jcb.200709076.

[3] J. Y. Tinevez, N. Perry, J. Schindelin, G. M. Hoopes, G. D. Reynolds, E. Laplantine, S. Y. Bednarek, S. L. Shorte, K. W. Eliceiri, *Methods* **2017**, *115*, 80, https://doi.org/10.1016/j.ymeth.2016.09.016.

[4] F. Coussy, L. de Koning, M. Lavigne, V. Bernard, B. Ouine, A. Boulai, R. El Botty, A. Dahmani, E. Montaudon, F. Assayag, L. Morisset, L. Huguet, L. Sourd, P. Painsec, C. Callens, S. Chateau-Joubert, J. L. Servely, T. Larcher, C. Reyes, E. Girard, G. Pierron, C. Laurent, S. Vacher, S. Baulande, S. Melaabi, A. Vincent-Salomon, D. Gentien, V. Dieras, I. Bieche, E. Marangoni, *International journal of cancer* **2019**, *145* (7), 1902, https://doi.org/10.1002/ijc.32266.

[5] A. Bruna, O. M. Rueda, W. Greenwood, A. S. Batra, M. Callari, R. N. Batra, K. Pogrebniak, J. Sandoval, J. W. Cassidy, A. Tufegdzic-Vidakovic, S. J. Sammut, L. Jones, E. Provenzano, R. Baird, P. Eirew, J. Hadfield, M. Eldridge, A. McLaren-Douglas, A. Barthorpe, H. Lightfoot, M. J. O'Connor, J. Gray, J. Cortes, J. Baselga, E. Marangoni, A. L. Welm, S. Aparicio, V. Serra, M. J. Garnett, C. Caldas, *Cell* **2016**, *167* (1), 260, https://doi.org/10.1016/j.cell.2016.08.041.
